# The terminal enzymes of (bacterio)chlorophyll biosynthesis

**DOI:** 10.1098/rsos.211903

**Published:** 2022-05-04

**Authors:** Matthew S. Proctor, George A. Sutherland, Daniel P. Canniffe, Andrew Hitchcock

**Affiliations:** ^1^ Plants, Photosynthesis and Soil, School of Biosciences, University of Sheffield, Firth Court, Western Bank, Sheffield S10 2TN, UK; ^2^ Biochemistry and Systems Biology, Institute of Systems, Molecular and Integrative Biology, University of Liverpool, Crown Street, Liverpool L69 7ZB, UK

**Keywords:** (bacterio)chlorophyll, chlorophyll synthase, ChlG, geranylgeranyl reductase, ChlP, photosynthesis

## Abstract

(Bacterio)chlorophylls are modified tetrapyrroles that are used by phototrophic organisms to harvest solar energy, powering the metabolic processes that sustain most of the life on Earth. Biosynthesis of these pigments involves enzymatic modification of the side chains and oxidation state of a porphyrin precursor, modifications that differ by species and alter the absorption properties of the pigments. (Bacterio)chlorophylls are coordinated by proteins that form macromolecular assemblies to absorb light and transfer excitation energy to a special pair of redox-active (bacterio)chlorophyll molecules in the photosynthetic reaction centre. Assembly of these pigment–protein complexes is aided by an isoprenoid moiety esterified to the (bacterio)chlorin macrocycle, which anchors and stabilizes the pigments within their protein scaffolds. The reduction of the isoprenoid ‘tail’ and its addition to the macrocycle are the final stages in (bacterio)chlorophyll biosynthesis and are catalysed by two enzymes, geranylgeranyl reductase and (bacterio)chlorophyll synthase. These enzymes work in conjunction with photosynthetic complex assembly factors and the membrane biogenesis machinery to synchronize delivery of the pigments to the proteins that coordinate them. In this review, we summarize current understanding of the catalytic mechanism, substrate recognition and regulation of these crucial enzymes and their involvement in thylakoid biogenesis and photosystem repair in oxygenic phototrophs.

## 1. Introduction

Chlorophylls (Chls) are modified tetrapyrroles synthesized by photosynthetic organisms and are critical to the primary reaction of photosynthesis, the harvesting of light energy to drive an electron transport chain and produce ATP and NADPH for carbon fixation. Chls absorb light in the blue and red region of the electromagnetic spectrum and absorb poorly in the green region, giving oxygenic phototrophs—plants, algae and cyanobacteria—their distinctive green colour [1]. The Chls that perform this absorption are organized within intricate protein–pigment assemblies, known collectively as light-harvesting antenna complexes, in a configuration that permits the transfer of light energy between neighbouring Chls. Light energy is ultimately funnelled to photosystem complexes where a ‘special pair’ of redox-active Chl molecules facilitate charge separation, essentially converting light energy into chemical energy. As such, Chl is responsible for supplying a large portion of the biosphere with life-sustaining energy.

**Figure 1.**
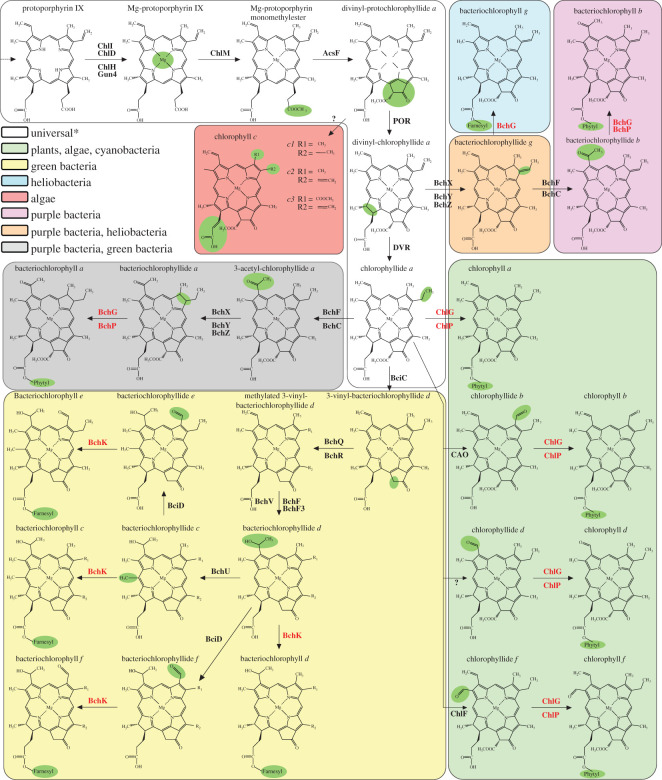
(Bacterio)chlorophyll biosynthesis pathways in photosynthetic organisms. The first steps of (bacterio)chlorophyll ((B)Chl) biosynthesis are shared between all photosynthetic organisms and involve chelation of a magnesium ion at the centre of the porphyrin ring and formation of the characteristic ring E producing divinyl-protochlorophyllide *a* (DV-PChlide *a*). Reduction of ring D of DV-PChlide *a* produces divinyl-chlorophyllide *a*, the last common precursor in plants, algae* and cyanobacteria, purple bacteria, heliobacteria, and ‘green bacteria’ (green sulfur bacteria, filamentous anoxygenic phototrophs (FAPs) and Acidobacteria). *Note that in some algal species variants of Chl *c* are produced from DV-PChlide *a* by action of an unknown enzyme(s) and differ depending on the identity of the ring B side chains (R1 and R2). Further specific modifications that produce the various species of (B)Chl are colour-coded according to the organism(s) in which they are synthesized along with the enzymes that catalyse the reactions. Except for Chls *c*, Chl biosynthesis and the biosynthesis of BChls *a* and *b* terminates with the esterification and reduction of a C20 isoprenoid moiety to ring D by ChlG/BchG and ChlP/BchP, respectively. A C15 farnesyl tail is typically added to BChlides *c*, *d*, *e*, *f* and *g* by BchK (BChls *c–f*) or BchG (BChl *g*). Note that some reactions may occur in alternative orders, as detailed in the text.

**Figure 4.**
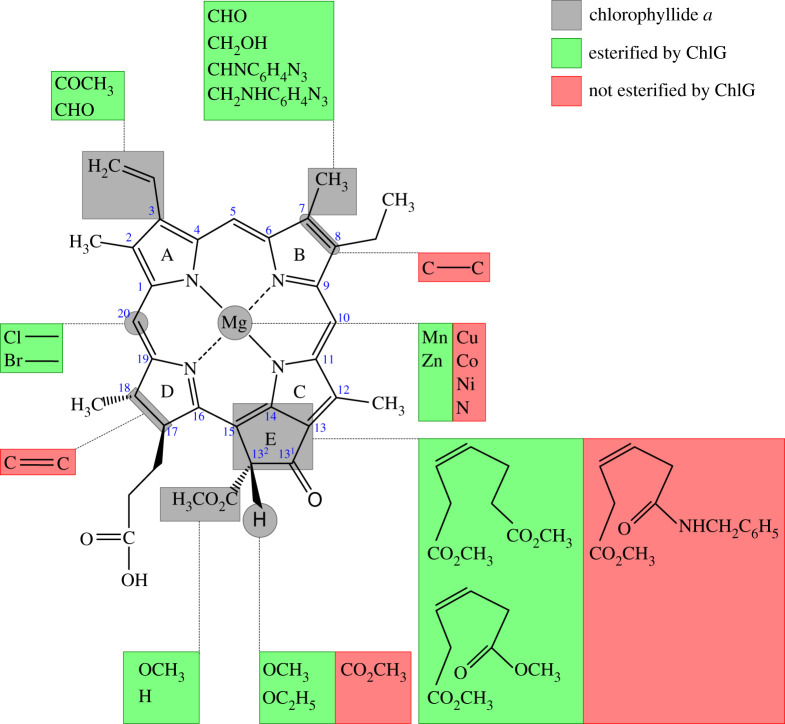
Reactivity of modified tetrapyrrole compounds with chlorophyll synthase (ChlG). Chemical modification of chlorophyllide *a* (grey) to produce compounds that can be esterified by ChlG are highlighted in green and those that can no longer act as a substrate in red. ChlG requires reduction of ring D but that ring B remain oxidized, chelation of a central metal ion that forms a pentacoordinate square-pyramidal conformation, and that no bulky substituents occupy side chain positions around ring E. Modification of ring A and B side chains are tolerated. Carbon atom numbering around the tetrapyrrole macrocycle is indicated in blue.

Chls share the same basic structure, consisting of four pyrrole rings (A–D) arranged in a macrocycle that coordinates a central Mg^2+^ ion, and a modification to give an archetypal fifth isocylcic ‘E’ ring. Seven major forms of Chl, lettered *a*, *b*, *c1*, *c2*, *c3, d* and *f*, have been discovered to date (figure 1) and differ in the functional groups located at positions C2, 3, 7, 8 and 17 (see figure 4 for macrocycle nomenclature). Chls *c1*, *c2* and *c3* also lack an isoprenoid moiety esterified to the C17 position on ring D. Bacteriochlorophylls (BChls) are synthesized by anoxygenic phototrophs and differ from Chls primarily in the reduction state of the C17-18 bond (in the case of the true BChls) along with other differences in pyrrole functional groups (figure 1).

The most abundant Chl species is Chl *a*, which is found in all oxygenic phototrophs. Biosynthesis of Chl *a* consists of 17 enzymatic steps catalysed by 15 enzymes and shares a pathway with heme biosynthesis up until the point of metal ion insertion into protoporphyrin IX; magnesium chelatase chelates future Chl molecules with Mg^2+^ [2] whereas ferrochelatase inserts Fe^2+^ to make heme B [3]. Following Mg^2+^ insertion, the reactions of the enzymes Mg-protoporphyrin methyltransferase [4] and Mg-protoporphyrin monomethylester cyclase [5] form the characteristic E ring of the chlorin macrocycle producing divinyl-protochlorophyllide (DV-PChlide). Next, PChlide oxidoreductase (POR) reduces the C17 = C18 double bond of ring D [6,7] and divinyl reductase (DVR) reduces the vinyl group at the C8 position of ring B to an ethyl group [8,9], resulting in the production of monovinyl-chlorophyllide (Chlide *a*); note that these reactions may occur in either order. Chl *a* biosynthesis concludes with the attachment of a reduced C20 isoprenoid alcohol to the C17 propionate side chain of ring D of Chlide *a* by the action of two enzymes, geranylgeranyl reductase (GGR; ChlP) [10] and chlorophyll synthase (ChlG) [11]. In some organisms, Chl/Chlide *a* can be modified to Chl/Chlide *b* (formyl group at C7), Chl/Chlide *d* (formyl rather than vinyl group at C3) or Chl/Chlide *f* (formyl group at C2) (figure 1). The Chl *f* synthase (ChlF; [12]) and Chl/Chlide *a* oxygenase (CAO, for Chl *b* synthesis; [13]) have been identified, but the enzyme(s) responsible for Chl *d* synthesis is unknown. Schliep *et al*. [14] suggest the C3 modification of Chl *d* occurs after tail addition, but it is not clear if ChlF and CAO act before (i.e. on Chlide *a*) or after (i.e. on Chl *a*) esterification of the macrocycle by ChlG. BChls are also synthesized by modification of (DV-)Chlide *a,* with the reduction of the C7-C8 double bond by the enzyme Chlide *a* oxidoreductase (COR) converting the chlorin ring to a bacteriochlorin ring in the case of BChls *a*, *b* and *g*, or removal of the C13^2^ methylcarboxyl group from ring E by Chlide *a* hydrolase (BciC) in the case of BChls *c–f*. For further details on (B)Chl biosynthesis we refer the reader to a recent review of tetrapyrrole biosynthesis [15].

The integral thylakoid membrane protein ChlG, and its homologue in BChl containing organisms, BChl synthase (BchG), are members of a family of enzymes called polyprenyl transferases. These enzymes are responsible for the esterification of (B)Chlide with an isoprenoid alcohol, in the majority of cases a C20 molecule of geranylgeraniol from geranylgeranyl pyrophosphate (GGPP) or phytol from phytyl pyrophosphate (PPP) [16]. Esterification with GGPP produces geranylgeranyl-chlorophyll (GG-Chl), which is subsequently reduced to a phytyl moiety by the action of the membrane-associated enzyme ChlP (BchP in anoxygenic phototrophs) [10]. ChlP uses NADPH to perform three consecutive reductions of the GG moiety, producing two intermediary species, dihydro-geranylgeranyl (DHGG)-Chl (one reduction) and tetrahydro-geranylgeranyl (THGG)-Chl (two reductions) [17,18]. Alternatively, ChlP can reduce free GGPP to PPP prior to ChlG catalysis [16]. Although addition of an isprenoid tail has no spectroscopic effect on the chlorin ring, the hydrophobicity of the pigment is significantly enhanced, which aids the binding and positioning of Chl within membrane-intrinsic photosynthetic protein complexes [19]. In this respect, ChlG operates at the interface between the pathways of Chl biosynthesis and thylakoid membrane biogenesis, partaking in Chl handover to the photosystem assembly/repair apparatus [20]. In this review, we summarize the literature pertaining to the final steps of Chl and BChl biosynthesis, highlighting gaps in our current knowledge, and discuss unresolved questions to be tackled by future research.

## 2. Discovery of (bacterio)chlorophyll synthases in photosynthetic organisms

The esterification of Chlide was first reported in 1911, when Willstatter and Stoll discovered hydrolysis of Chl to Chlide in crude extracts of *Heracleum* leaves and named the enzyme responsible for this reaction chlorophyllase. This enzyme also exhibited limited activity in the opposite direction, esterification of Chlide with PPP to form Chl [21,22]. Over the following years, examination of the *in vitro* esterification activity of algal and plant chlorophyllases produced conflicting results [23–26]. Although chlorophyllase appeared capable of esterifying various species of Chlide with differing tetraprenyl moieties [27,28], the enzyme invariably demonstrated a requirement for high concentrations of Chlide and organic solvent in order to shift the reaction equilibrium in favour of esterification [29]. Nevertheless, chlorophyllase was postulated to participate in the final step of the Chl biosynthesis pathway in young plants by esterifying Chlide before catalysing the opposite reaction and breaking down Chl in mature plants [26,29]. However, the extreme assay conditions required to promote esterification activity prompted the suggestion that another enzyme was responsible for Chl synthesis *in vivo* [30–32].

Rüdiger *et al*. [33] found the first evidence that an enzyme other than chlorophyllase catalyses esterification of Chlide. ^14^C-labelled GGPP was esterified to Chlide when incubated with maize shoot extracts in the absence of organic solvents. The solvent-independent activity of this enzyme was further demonstrated in oat seedlings in which 80–90% of total Chlide was esterified [11]; by comparison, in previous experiments chlorophyllase converted just 1–15% of Chlide to Chl [31,32]. Furthermore, Rudoı˘ *et al*. [34] showed production of Chl from exogenous Chlide upon incubation with leaf extract, but not production of pheophytin (Chl lacking the central Mg^2+^ ion) from pheophorbide (Chlide lacking Mg). Conversely, addition of Chl and pheophytin resulted in production of Chlide and pheophorbide, respectively, demonstrating the existence of two distinct enzymes. Accordingly, the enzyme responsible for esterification of Chlide was named Chl synthase in order to distinguish it from chlorophyllase [11].

BchG was identified in the purple bacterium *Rhodobacter* (*Rba*.) *capsulatus* by site-directed mutagenesis of *orf304* in the photosynthesis gene cluster, producing a mutant that failed to synthesize BChl *a* and accumulated bacteriochlorophyllide (BChlide) *a* [10]. This discovery was followed by identification of *bchG*/*chlG* homologues in the genomes of other model photosynthetic organisms including the purple bacteria *Rba*. *sphaeroides* [35] and *Rhodospirillum* (*Rsp.*) *rubrum* [36], the cyanobacterium *Synechocystis* sp. PCC 6803 (hereafter *Synechocystis*) [37,38], the plants *Arabidopsis thaliana* [39] and *Oryza sativa* (rice) [40], and the ‘green bacteria’ *Chloroflexus* (*Cfx*.) *aurantiacus* [41] and *Chlorobium* (*Cba*.) *tepidum* [42]. In most cases, evidence that these genes encoded BchG or ChlG was demonstrated by heterologous production in *Escherichia coli* and assaying cell lysates for enzyme activity by detection of Chl/BChl after exogenous addition of substrates [35,40,43,44].

## 3. Chlorophyll synthase activity in oxygenic phototrophs

ChlG is an essential enzyme in oxygenic phototrophs such as plants, algae and cyanobacteria, and its activity is associated with the thylakoid membranes of cyanobacteria [20,45–47] and mature plant chloroplasts [48–50]. Immunoprecipitation experiments with tagged ChlG from *Synechocystis* have shown that the protein co-purifies with YidC and ribosomal subunits, indicating that it is probably translated by membrane-bound ribosomes and co-translationally inserted into the membrane by the SecYEG translocon with the assistance of the YidC membrane-insertase [20,45].

In plants, ChlG is encoded by a single gene (*chlG*) that is constitutively expressed at low levels in all green tissues throughout growth [39,40,51]. ChlG activity is specifically associated with the inner membrane fractions of young plant etioplasts, consisting of prolamellar bodies (PLBs) and prothylakoids (PTs) [11,52–56]. An initial study suggested the enzyme is inactive within intact PLBs [56] until their light-induced conversion to PTs, which correlates with the ‘Shibata shift’ (a change in the absorption maximum of Chlide from 684 to 672 nm) and the light-dependent production of Chlide by POR; ChlG activity increases as the enzyme and its substrates are transferred from the PLB fraction to the newly forming PTs during plant greening [55,56].

Subsequently, two phases of Chlide esterification have been described in etiolated leaves. A pulse of light initiated a fast phase of esterification that converted 15% of the Chlide pool into Chl during the first 15–30 s after illumination. This was followed by a lag phase of approximately 2 min, before initiation of a second slow phase (30–60 min) during which the remaining Chlide is phytylated [57,58]. The fast phase of esterification always resulted in the same quantity of Chl production and was unaffected by the light intensity, Chlide availability, low temperature or the disaggregation of PLBs, and could be restored by a period of darkness before a second pulse of light. By contrast, the slow phase is abolished at low temperatures and is dependent upon the disaggregation of the PLBs [58].

The two phases of Chlide esterification by ChlG are thought to be the result of several factors including: (i) the disaggregation of PLBs; (ii) the rate of diffusion of GGPP/PPP in the membrane bilayer; (iii) the Shibata shift, which has been linked to the release of Chlide from POR ternary complexes and the transfer of the ternary complexes from the PLBs to PTs; and (iv) the pre-loading of ChlG with GGPP/PPP [58–65]. The fast phase correlates with the rapid conversion of a pool of POR-bound photoactive PChlide to Chlide upon exposure to light. In this scenario, the release of Chlide is immediately followed by replacement with PChlide due to the high concentration of the latter in PLBs. Thus, in the fast phase, replacement of Chlide with PChlide precedes rebinding of NADPH to POR [66].

**Figure 2.**
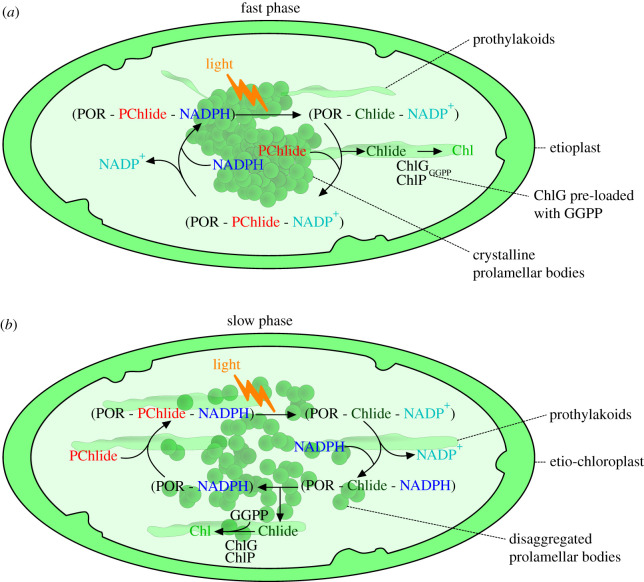
Model of the fast and slow phases of chlorophyll production by ChlG and ChlP. The model is based on data from the references described in the text. (*a*) Exposure of etioplasts to light activates a ternary complex consisting of protochlorophyllide reductase (POR; black), protochlorophyllide (PChlide; red) and NADPH (blue). POR catalyses electron transfer from NADPH to PChlide, producing NADP^+^ (cyan) and chlorophyllide (Chlide; dark green), respectively. A high concentration of PChlide in the prolamellar bodies (PLBs) promotes replacement of Chlide with PChlide in the ternary complex; newly released Chlide is subsequently esterified to GGPP, which is already bound to ChlG (ChlG_GGPP_), followed by reduction of the GG tail to phytyl by ChlP, forming chlorophyll (Chl; green). NADPH replaces NADP^+^ and the POR cycle repeats. (*b*) The ensuing slow phase of Chl formation becomes prominent as the PLBs disaggregate in etio-chloroplasts and prothylakoid formation increases. PChlide becomes limiting and Chlide is not immediately released from the POR ternary complex. Instead, NADP^+^ replacement by NADPH precedes release of Chlide, thus, the flux of the Chlide substrate towards ChlG/ChlP is decreased and Chl formation slows; this is further accentuated by the need for rebinding of GGPP to ChlG.

The released Chlide is esterified by nearby ChlG, as shown in figure 2*a* [57,66]. This model assumes pre-loading of ChlG with GGPP/PPP [58,64] and the possible association of ChlG with POR [58], although the latter has yet to be conclusively demonstrated [66]. The essential nature of GGPP/PPP pre-loading was demonstrated by the fact that sodium fluoride treatment, which inhibits the slow phase of Chlide esterification, presumably by preventing GGPP/PPP production, did not affect the fast phase [65], indicating that if the enzyme already contains GGPP/PPP then Chlide binding upon illumination can proceed unperturbed.

The second phase of esterification is comparatively slow due to the need for rebinding of GGPP to ChlG [56–58]. Also implicated in the slow phase is the rate of disaggregation of POR complexes, concurrent with the Shibata shift, and the changes in cellular architecture during the dispersal of PLBs and the formation of PTs [60,61,63]. The concentration of PChlide is more limiting under these conditions and so newly formed Chlide is not immediately released from POR; instead, replacement of NADP for NADPH occurs before Chlide is released [66] (figure 2*b*).

The fast and slow phases of Chl formation have also been investigated *in vitro* where ChlG was produced in *E. coli* and cell lysates were pre-incubated with PPP. Upon addition of exogenous Chlide, the lysates exhibited a rapid phase of esterification that lasted 15–30 s, comparable to that in leaves [64]. These results support a model in which pre-loading of ChlG with GGPP/PPP enables a rapid phase of Chl formation and the subsequent slow phase is limited by the availability and rate of diffusion of further GGPP/PPP substrate in the lipid bilayer [64].

During the later stages of plant growth, ChlG activity remains associated with the thylakoid fraction of mature plant chloroplasts [48–50]. Interestingly, the activity of ChlG appears to be enhanced within crude chloroplast extracts in which the thylakoid and stromal fractions are not separated [48]. This could be because the stromal fraction contains essential cofactors that enhance ChlG activity, or may reflect an increased efficiency of substrate delivery from preceding stromal Chl biosynthesis enzymes to membrane-bound ChlG. ChlG activity has also been associated with the stromal fraction of spinach chloroplasts [50] and daffodil chromoplasts [67]. In the latter case, ChlG activity was confined to the stromal fraction, which esterified 66% of exogenously added Chlide [67]. It has been argued that these results are an artefact of the isolation methods used to prepare chloroplast fractions [50], or that esterification activity within chromoplasts, which do not contain Chl, is due to an as-yet unidentified membrane-associated enzyme rather than the membrane-integral ChlG [67].

## 4. Synthesis of bacteriochlorophylls *a*, *b* and *g* in anoxygenic phototrophs

The synthesis of BChls is best studied in purple phototrophic bacteria such as *Rba. sphaeroides*, which induce pigment biosynthesis in response to anoxic conditions and light [68,69]. The pathway of BChl *a* biosynthesis in anoxygenic phototrophs is analogous to that of Chl *a* in oxygenic phototrophs up to the synthesis of Chlide *a* [15]. Following this, two additional modifications of Chlide *a* result in the production of BChlide *a*: (i) the C7 = C8 double bond of ring B is reduced by COR (BchXYZ) to produce 3-vinyl-BChlide *a* (3V-BChlide *a*) [70,71]; and (ii) 3V-BChlide *a* is converted to BChlide *a* by conversion of the C3 vinyl group to an acetyl group by the activities of 3V-BChlide hydratase (BchF) and BChlide dehydrogenase (BchC) [72–74]. Note that BchF and BchC may act before COR (producing 3-acetyl-Chlide *a*), as shown in figure 1. Subsequently, GGPP is esterified to BChlide *a* by BchG and reduced by BchP, or BchP may reduce GGPP to PPP prior to esterification by BchG [10,75,76]. In the synthesis of BChls *b* and *g*, COR acts on DV-Chlide *a* to produce 3V-BChlide *b* (also referred to as BChlide *g*), which has a ethylidene group at C8 [77,78]. In BChl *b* synthesis, BChlide *b* is formed from 3V-BChlide *b* by BchF and BchC (figure 1). BchG adds a farnesyl pyrophosphate (FPP) or GGPP/PPP tail to BChlide *g* or BChlide *b*, respectively, producing BChl *g* or BChl *b*.

Genetic studies showed that *bchG* encodes BChl synthase in *Rba. capsulatus* [10] and *Rba. sphaeroides* [75]; both enzymes have been produced in *E. coli* and tested for esterification activity by addition of BChlide and GGPP/PPP to cell-free lysates [35,44]. BchG has also been identified in the green sulfur bacterium *Cba*. *tepidum* [79], the filamentous anoxygenic phototroph (FAP) *Cfx*. *aurantiacus* [41] and *Heliobacteria* [78].

## 5. Geranylgeranyl reductase

**Figure 3.**
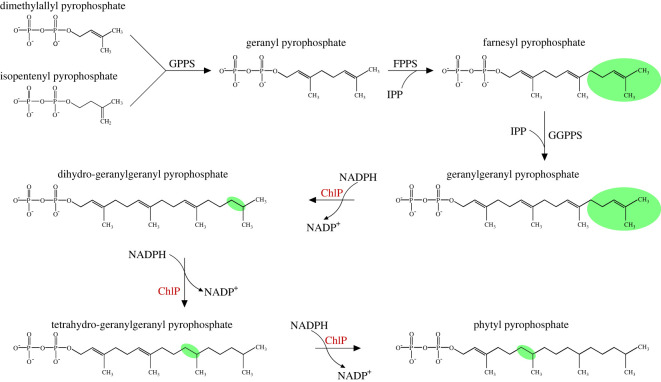
Phytyl pyrophosphate biosynthesis in oxygenic phototrophs. Dimethylallyl pyrophosphate (DMAPP) and isopentenyl pyrophosphate (IPP) are condensed to form geranyl pyrophosphate (GPP) and condensation of GPP with two further molecules of IPP produces farnesyl pyrophosphate (FPP) and then geranylgeranyl pyrophosphate (GGPP). A series of three consecutive reductions of GGPP by ChlP, using NADPH as a reductant, generates phytyl pyrophosphate (PPP) via the intermediates dihydro-GGPP and tetrahydro-GGPP.

GGPP is a critical precursor in several vital metabolic pathways in both eukaryotes and prokaryotes, where in addition to (B)Chl it is required for synthesis of terpenoids including carotenoids, plant hormones, gibberellins, quinones, tocopherols, lipids and dolichol [80–83]. GGPP is synthesized by the condensation of isopentenyl pyrophosphate (IPP) and dimethylallyl pyrophosphate (DMAPP) and subsequent condensation with two additional IPP molecules (figure 3) [84–86], and represents an important metabolic hub where the flux of GGPP into various metabolic pathways is controlled [82,87–89]. The enzymes responsible for catalysing this reaction are known as GGPP synthases (GGPPS). Members of this diverse family associate with downstream metabolic enzymes to ensure substrate channelling into the appropriate pathways. In plant chloroplasts, one such GGPPS co-localizes with a GGPPS-recruiting protein (GRP) in the thylakoid membrane [89].

ChlP and BchP catalyse the NADPH- and ATP-dependent reduction of three of the four C=C double bonds of GGPP to produce PPP [10] (figure 3). Genes encoding ChlP/BchP have been identified in oxygenic phototrophs including cyanobacteria [90] and plants [17,18,91,92], as well as in purple bacteria [76] and green bacteria [93,94]. Deletion of *chlP* from *Synechocystis* results in the accumulation of Chl molecules with unreduced tail moieties that are incorporated into Chl-binding proteins and can still function in light harvesting. Photosystem I (PSI) and photosystem II (PSII) are functional in these mutants, but photoautotrophic growth is abolished due to the rapid degradation of the photosystems [16,95]. The same growth phenotype is observed in plants lacking *chlP* [18,96], which also display an increased sensitivity to high-light stress [97]. The reduced stability of the photosystems induced by integration of unreduced Chls has been attributed to the increased rigidity of GG-Chl, which may disrupt the assembly of the complexes [16,95]. Reduction of the Chl tail also appears to be important for mediating the interactions between neighbouring Chl molecules, enabling the efficient transfer of absorbed light energy to the photosystem reaction centre [95]. Although purple bacteria harbouring GG-BChl *a* had reduced reaction centre stability [10], they were able to grow photoautotrophically, albeit at a slower rate than the wild-type strains [76]. Note that in *Rsp. rubrum*, which produces GG-BChl *a*, the BchP enzyme is specific for the reduction of the isoprenoid moiety of bacteriopheophytin (BPheo) *a* [36], while the BchP enzymes of some other bacteria miss out one reduction of GGPP resulting in (B)Chls esterified with Δ2,6-phytadienyl or Δ2,10-phytadienyl TH-GG tails (see below).

## 6. Substrate specificity of ChlG/BchG for tetraprenyls

Two pathways of Chl esterification have been described in photosynthetic organisms. One consists of esterification of Chlide with GGPP by ChlG, followed by the stepwise reduction of the GG moiety by ChlP to produce mature Chl *a*; alternatively, ChlP can act on free GGPP to produce PPP that is used by ChlG to esterify Chlide [10,16]. The former pathway appears to predominate in the etioplasts of young plants and the chloroplast envelope of mature plants [11,50,58,98,99], whereas the latter occurs in the thylakoid membranes of mature greening plants [50,100]. The reasons for this remain unclear; however, it is likely that utilization of either GGPP or PPP by ChlG is dependent upon the availability of each substrate, which in turn is contingent on the varying levels of ChlP during plant growth [50,101,102]. ChlP production is upregulated during chloroplast development, thus, GGPP may be more readily available in the etioplasts of young seedlings [17], whereas in mature plants PPP prevalence increases with ChlP abundance and GGPP availability may decrease due to demand from the carotenoid biosynthesis pathway [50].

A small population of Chls are esterified with a shorter farnesyl moiety (Chl *a*_f_) in some species of thermophilic cyanobacteria. These Chl *a*_f_ molecules appear to specifically localize within the CP43 subunit of the PSII complex [103]. Esterification of Chlide with FPP is presumably catalysed by ChlG; indeed, ChlG has been shown to use exogenous FPP as a substrate [11].

In anoxygenic phototrophs, BChl *a* is almost universally found in the phytylated form [104,105]. Upon the onset of BChl *a* biosynthesis, some purple bacteria accumulate three minor BChl species, GG-BChl, DH-GG-BChl and TH-GG-BChl, in addition to BChl *a* [106,107]*,* indicating that BchG can act before BchP. This is reinforced by the observation that *Rsp. rubrum* synthesizes unreduced BChl *a*_GG_, but also incorporates the de-metallated BPheo *a* with a phytyl tail into its reaction centre; the BchP enzyme from this organism is only able to reduce the tail of BPheo, indicating that BchG must esterify BChlide with GGPP, and that this pigment undergoes Mg-dechelation before the isoprenoid moiety is reduced [36,105]. An additional unique case is that of the anoxygenic phototrophic bacterium *Halorhodospira halochloris*, which accumulates BChl *b* carrying a tail with only two reductions (a 2,10-phytadienyl moiety), thought to be a result of an insertion mutation in the *bchP* gene [108,109]. Furthermore, *Cba*. *tepidum* produce BChl *a* with a phytyl tail, but also Chl *a* with a unique TH-GG group with two reductions (a 2,6-phytadienyl moiety) [108,110].

ChlG from *Synechocystis*, produced recombinantly in *E. coli*, preferred PPP to GGPP [44], whereas the enzymes from *A*. *thaliana* [43] and *Avena sativa* (oat) [64] showed a preference for GGPP. Similarly, BchG homologues from *Cba. tepidum* [79] and *Cfx. aurantiacus* [111] preferentially esterified BChlide with GGPP over PPP (and FPP) when assayed in *E. coli* lysates; the *Cba*. *tepidum* BchG used FPP more efficiently during the first 5 min of the reaction before preferentially consuming GGPP. Conversely, BchG from *Rba. capsulatus* showed a preference for PPP in a recombinant system [44]. Like ChlG, BchG from *Rba. sphaeroides* has also been shown to use FPP as well as GGPP both *in vitro* and *in vivo* [78,79].

GGPP appears to be rapidly metabolised by *E. coli* cell lysates to a form that is no longer a substrate of ChlG [64]. Given this, the substrate specificity of ChlG for its isoprenoid substrates requires *in vivo* kinetics in the native organism [58] or enzyme assays using purified ChlG. Chidgey *et al*. [20] demonstrated such an assay using a *Synechocystis* ChlG protein complex isolated by FLAG immunoprecipitation in detergent and the exogenous addition of GGPP and Chlide.

Despite conflicting reports concerning the preferential reactivity of ChlG with GGPP or PPP *in vivo* compared with *in vitro*, it is clear that ChlG requires diphosphorylated isoprenoids for activity. ChlG from plant etioplasts was able to incorporate GGPP, PPP and FPP into Chlide in the absence of exogenous ATP, whereas the monophosphorylated derivatives of these compounds were only partially esterified, and the unphosphorylated variants not at all unless ATP was provided [11]. Similar experiments performed with ChlG produced in *E. coli* demonstrated that unphosphorylated substrates were not accepted, even in the presence of ATP, indicating that plant etioplasts produce a kinase that is absent in *E. coli* [51].

## 7. Tetrapyrrole substrate specificity of ChlG/BchG

By contrast to the apparently relatively loose specificity of ChlG and BchG for the isoprenoid tails, both enzymes exhibit more specific recognition of their tetrapyrrole substrates. Many tetrapyrrole compounds have been tested for reactivity with ChlG/BchG with the aim of determining the tolerance of the enzyme for variance in the central metal ion, chemical substituents of the pyrrole rings and the reduction state of the macrocycle (summarized in figure 4). Differences between Chls and BChls are mainly in rings A and B. Chls typically have a vinyl group at C3 of ring A (formyl in Chl *d*), as does BChl *g*, whereas BChls *a* and *b* have an acetyl group at this position, Chls *b* and *f* have substitutions at C7 and C2, respectively, and BChls *b* and *g* have an ethylidene substituent in place of the ethyl group at the C8 position. However, it appears that the predominant determinant of substrate specificity by ChlG and BchG is whether the ring B C7-C8 bond is reduced, as is the case in true BChls, or oxidized, as in Chls.

In addition to a central metal ion that can form a pentacoordinate square-pyramidal conformation and reduction of the C17-C18 double bond of ring D, ChlG enzymes appear to require that the C7-C8 double bond of ring B remains oxidized [34,51,52,64,112]. ChlG homologues do not esterify BChlide *a* (acetyl at C3 and C7-C8 bond reduced) [44] but can esterify Chlides *a*, *b*, *d* and *f* (*d* and *f* presumed but not definitively shown) and DV-Chlide *a*, which all contain a double C7-C8 bond but differ at the C2 (Chlide *f*), C3 (Chlide *d*), C7 (Chlide *b*) and C8 (DV-Chlide *a*) positions [34,64,113]. This indicates that exocyclic differences in the groups of ring A and B (figure 1) have less influence than the reduction state of the C7-C8 bond of the macrocycle in substrate recognition.

The structure of the substituents on ring E also seem to be important as Chlide *a*', where the C13^2^ methyl ester group and proton of ring E are orientated in the opposite direction to that of Chlide *a*, is not esterified by ChlG. This indicates that naturally occurring Chl *a*' must be formed by epimerization of Chl *a* following esterification of Chlide *a* [114]. Lack of the C13^2^ methyl ester had no effect on ChlG activity; however, replacing the C13 H with methoxy or ethoxy groups severely perturbed enzyme activity, suggesting the enzyme cannot tolerate bulky substituents at the C13 position [64].

Like ChlG, BchG requires a metal ion capable of forming a pentacoordinate square-pyramidal conformation at the centre of the macrocycle ring [44,111]. In addition to BChlide *a*, BchG from *Rba. sphaeroides* can esterify BChlide *b* and *g* [77,78], which have a reduced C7-C8 double bond but differ at the C8 positon, and in the case of BChlide *g* also at C3. However, the enzyme does not use substrates where the C7-C8 bond is unsaturated, including Chlide *a*, 3-hydroxyethylchlide *a* and BChlide *c* [115,116]. This allows BchG to differentiate BChlide *a* from Chlide *a*, which is produced as an earlier intermediate in the BChl biosynthesis pathway [44]. In addition, the Zn analogues of BChlides *c*, *d* and *e*, which despite their names are chlorins with a hydroxymethyl group at C3, are not substrates for BchG [111]; BChlides *c*, *d*, *e* and *f* are instead esterified by a third enzyme, bacteriochlorophyll *c* (*d*/*e*/*f*) synthase (BchK; discussed below).

ChlG and BchG exhibit competitive inhibition when provided with the ‘wrong’ substrate *in vitro*, suggesting that the active sites of the enzymes are similar [115]. In support of this, Kim *et al*. [116] identified a single residue that appears to be important in determining the substrate specificity of the enzymes. By producing cyanobacterial ChlG in a *bchG* deficient strain of *Rba. sphaeroides*, which cannot produce BChl, meaning the strain cannot photosynthesize, the authors isolated suppressor mutants that produced some BChl and were able to grow phototrophically. The suppressor mutation resulted in residue Ile44 in ChlG being substituted by Phe, which is found at the equivalent position in BchG homologues (see below). The I44F variant was able to use BChlide as a substrate when produced recombinantly, and the corresponding BchG F28I enzyme was able to esterify Chlide *in vitro*; in both cases the mutant enzymes worked on the ‘wrong’ substrate with significantly lower efficiency than their natural substrate, for which their affinity did not change. We have identified the same ChlG suppressor mutation in analogous experiments and introduced the F28I variant of BchG into *Synechocystis,* but the variant enzyme is not sufficiently active with Chlide as substrate to allow replacement of the native ChlG (Proctor and Hitchcock 2022, unpublished).

The *K*_i_ for the inhibition of ChlG by BChlide is increased approximately fourfold when PPP is bound to the enzyme instead of GGPP. It was suggested that the reduced bonds of PPP resulted in a decrease in BChlide binding to ChlG, perhaps by inducing structural changes to ChlG [115]. Conversely, pre-binding of PPP or GGPP did not increase the *K*_i_ value for inhibition of BchG by Chlide. This is interesting when considering that Chlide *a* is an intermediate of the BChl biosynthesis pathway, whereas ChlG does not encounter BChlide in oxygenic phototrophs. The authors speculated that inhibition of BchG by Chlide is prevented by low intracellular concentrations of the latter and/or substrate channelling between earlier BChl biosynthesis enzymes, preventing leakage of Chlide into the photosynthetic membrane where BchG is situated [115].

## 8. Bacteriochlorophyll *c, d, e* and *f* synthases

In addition to BChl *a*, green sulfur bacteria and FAPS produce BChl *c* as their main pigment, as well as BChls *d* and *e* [117–121]. Despite the universally accepted BChl prefix, these molecules are classified as chlorins due to reduction of only the D ring and are therefore distinct from the true bacteriochlorins, BChls *a*, *b* and *g*, where ring B is also reduced [122,123]. BChls *c*, *d*, *e* and *f* (the latter of which is not produced naturally) share Chlide *a* as a common precursor, which undergoes specific modifications of its pyrrole side chains prior to esterification, primarily with FPP, by BchG isoforms collectively known as BchK (previously BchG2) (figure 1) [79,111,124,125]. Furthermore, some species also produce Chl *a* with a 2,6-pytadienyl tail (reduced only twice) and contain ChlG as a third esterifying enzyme. For a detailed review of (B)Chl biosynthesis in green bacteria see [93].

BchK was identified as a paralog of BchG in *Cfx. aurantiacus* and named BchG2 [111]. Deletion of the equivalent gene from *Cba*. *tepidum* prevented accumulation of BChl *c*, whereas BChl *a* and Chl *a* production was unaffected, confirming that BchG2 encodes BChl *c* synthase and prompting renaming of the enzyme as BchK [124]. Deletion of the gene *bchU*, encoding a C-20 methyltransferase required for synthesis of BChlide *c* and *e*, resulted in a *Cba*. *tepidum* strain that accumulated only BChl *d*, so BchK must also be able to esterify BChlide *d* [126]. Esterification of BChlides *e* and *f*, which differ from BChlides *c* and *d* at the C7 group, have been attributed to a second, recently identified clade of BchK named BchK2 [125].

Green sulfur bacteria contain several types of BChl *c* molecules esterified with various long-chain alcohols, indicating BchK exhibits promiscuous recognition of the isoprenoid substrate [79,127–133]. BChl *a* with a farnesyl tail is not detected in *Cba*. *tepidum*, despite the fact that BchG recognizes FPP as a substrate, possibly due to spatial separation of the sites of GGPP and FPP esterification by BchG and BchK, respectively [79]. BchK enzymes do not use Chlide *a* or BChlide *a* as substrates but the enzyme is active with the Zn analogues of BChlides *c* and *d in vitro*; both contain an oxidized C7-C8 bond and a C3 acetyl group, which appear to be essential for recognition by BchK [111]. In *Chloroflexus*-type green bacteria, BChl *c* is mainly esterified with stearol [93], and other esterifying fatty alcohols can be incorporated when supplied exogenously in the growth medium [128,134].

## 9. The structure of chlorophyll and bacteriochlorophyll synthases

Although a high-resolution structure of a ChlG, BchG or BchK enzyme is yet to be published, all are integral membrane proteins predicted to contain six to nine transmembrane helices (TMH) and with an approximate size of 30–40 kDa [41,51,64]. Considering the high degree of similarity in the amino acid sequences between (B)Chl synthases from various photosynthetic organisms, it is likely that these enzymes contain the same number of TMHs; the average number predicted for ChlG and BchG is eight [135] although structural models suggest that they contain nine (see below). The high degree of homology between ChlG, BchG and BchK enzymes implies restrictions to the structure throughout evolution, probably due to the well-defined spatial structure required to bind two amphiphilic substrates [51].

Structural characterization of proteins by methods such as X-ray crystallography and cryogenic electron microscopy is dependent upon production and isolation of pure proteins in relatively high quantities. A small quantity of active ChlG has been purified from the thylakoid membranes of *Synechocystis* by solubilization of membranes in detergent followed by affinity purification of the enzyme [20,45–47]. However, purification of membrane proteins from native organisms is often limited by low abundance. *E. coli* has been used for the production of recombinant ChlG from *A. sativa* [51], *A*. *thaliana* [43], *O. sativa* [40] and *Synechocystis* [44], as well as BchG from the purple bacteria *Rba. sphaeroides* [35] and *Rba. capsulatus* [44] and the green sulfur bacterium *Cba*. *tepidum* [79]. The recombinant proteins are active in *E. coli* lysates but purification of the enzymes following detergent solubilization appears to result in loss of activity [43], hindering the use of this method for structural work.

In the absence of a structure of a ChlG, BchG or BchK enzyme, structure-function information has been restricted to modelling and analysis of primary sequences, which has enabled identification of conserved residues and domains by comparison to other well-characterized members of the polyprenyltransferase family. Such comparisons indicate that the Mg^2+^ ion at the centre of Chlide is coordinated by an unknown residue in ChlG [112]. This may be indirectly via a second metal ion bound within the active site, similar to other polyprenyltransferases [41,136–138], or by a water molecule, which has been shown to coordinate the central Mg^2+^ within crystalline ethylchlorophyllide [139]. Polyprenyltransferase enzymes possess three conserved domains termed I, II and III. These domains have been identified in ChlG, BchG and BchK; in particular, domain II is highly conserved between the synthases and is implicated in binding of Mg^2+^ via a conserved DDXXD motif [39,41,136–138]. Alignment of ChlG and BchG orthologues required modification of this motif to DRXXD [41], although NDXXD, which occurs slightly earlier in the sequence (NDXXDRXXD; the underlined Asp is common to both motifs), could also be responsible for magnesium coordination [51]. In either case, these acidic motifs are suggested to interact with the negatively charged phosphates of polyprenyl diphosphates by proxy via coordination of Mg^2+^ ions, similar to the DRXXD motif of isoprenyl diphosphate synthases [140,141]. Alternatively, Arg or Lys residues could facilitate the interaction with the diphosphate groups of PPP/GGPP, as is the case in farnesyltransferases [142].

Site-directed mutagenesis of ChlG/BchG has progressed the structure–function understanding of the enzymes by enabling identification of features and residues essential for substrate binding and specificity. Schmid *et al*. [64] characterized domain II of the oat ChlG, predicted to lie within a loop between the second and third TMH. Mg^2+^ was essential for ChlG activity *in vitro*, indirect evidence that Mg^2+^ coordinates binding of PPP/GGPP. The authors identified an essential Arg residue (R161), proximal to the NDXXDRXXD region, which may be involved in GGPP/PPP binding. A follow-up study by the same group demonstrated that four residues within the motif (N146, D147, D150 and D154) are essential for enzyme activity, supporting the notion of diphosphate binding via complexed Mg^2+^ and promoting revision of the domain II motif to NDXXDRXXDXXXXXXR, starting with N146 and ending in the aforementioned R161 [64]. Schmid and colleagues also identified a Cys residue conserved in ChlG and BchG homologues (C109 in the oat enzyme) as essential for enzyme activity, and suggested it is involved in substrate binding or stabilization of the active form of the enzyme. N- and C-terminal truncation of the enzyme also revealed that the first 87 residues (which includes the chloroplast transit peptide) are dispensable for enzyme activity, although further deletion, which most likely interrupts the first TMH region, abolished catalysis. The enzyme could only tolerate removal of one residue from the C-terminus before loss of activity, again presumably due to disruption of the final TMH [51], which is in agreement with our unpublished data on the cyanobacterial enzyme.

**Figure 5.**
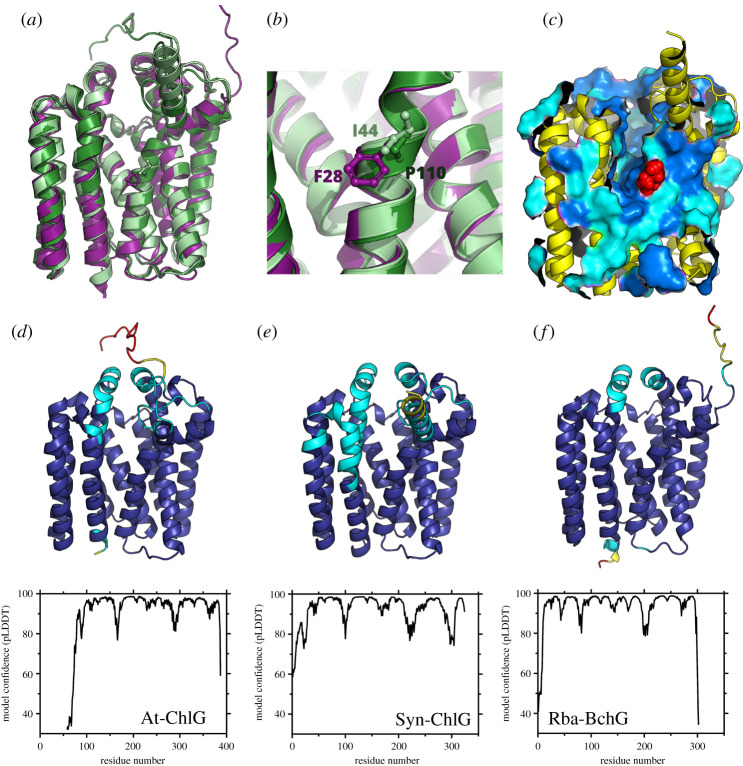
Computational models of (bacterio)chlorophyll synthases. (*a*) Overlay of ChlG from *A*. *thaliana* (At-ChlG, dark green), ChlG from *Synechocystis* (Syn-ChlG, light green) and BchG from *Rba. sphaeroides* (Rba-BchG, purple). (*b*) Magnified region of the three proteins showing differences in equivalent residues (P110 in At-ChlG, I44 in Syn-ChlG and F28 in Rba-BchG) that appear to be important for substrate specificity. (*c*) Partially surface rendered image of ChlG (yellow) to show conserved regions between all three proteins. Fully conserved residues are shown in dark blue and conservative changes in light blue. Residues with semi-conservative changes and no homology were not surface rendered. Red spheres indicate the location of the I44 residue. (*d–f*) Structural models (top) and pLDDT scores (bottom) are shown for At-ChlG (*d*), Syn-ChlG (*e*) and Rba-BchG (*f*). Residue colours correspond to the confidence thresholds set out in Tunyasuvunakool *et al*. [145], with high confidence (greater than 90 pLDDT) in dark blue, reasonable confidence (90–70) in cyan, low confidence (70–50) in yellow, and very low confidence (less than 50) in red. Note that the computational simulation of At-ChlG was run using the full-annotated sequence from the UniProt database but is displayed with the N-terminal chloroplast transit peptide removed.

Protein structure prediction by computation has recently experienced rapid advancement due to the application of machine-learning technology. One such prediction program, AlphaFold2, employs a deep learning artificial intelligence system to predict the three-dimensional structures of proteins [143,144]. Here we provide AlphaFold2 [144] structural models of ChlG from *Synechocystis* (Syn-ChlG) and *A*. *thaliana* (At-ChlG), and BchG from *Rba*. *sphaeroides* (Rba-BchG) (figure 5). All simulations were run on a NVIDA Tesla K80 GPU and conducted using the original monomer model with no ensembling and a PDB database threshold date of 1 January 2021. Displayed structures correspond to the model with the highest confidence score, calculated as the per-residue predicted local distance difference test (pLDDT) value of α-carbon atoms in the protein structure [146].

The three model structures were generated with a comparable degree of confidence and overlay closely with each other, with the exception of the first predicted TMH of Syn-ChlG (figure 5*a*). Interestingly, the position of the I44 (*Synechocystis*), F28 (*Rba. sphaeroides*) and P110 (*A. thaliana*) residues, which appear to be important for defining substrate specificity [116], are almost identical in each structure (figure 5*b*). These residues reside before a central cleft that would appear to correspond to the enzymes' active site, akin to the crystal structure of the related prenyl transferase enzyme UbiA [147]. The residues within this cleft are highly conserved between the three homologues, as highlighted by the coloured surface representation in figure 5*c*.

**Figure 6.**
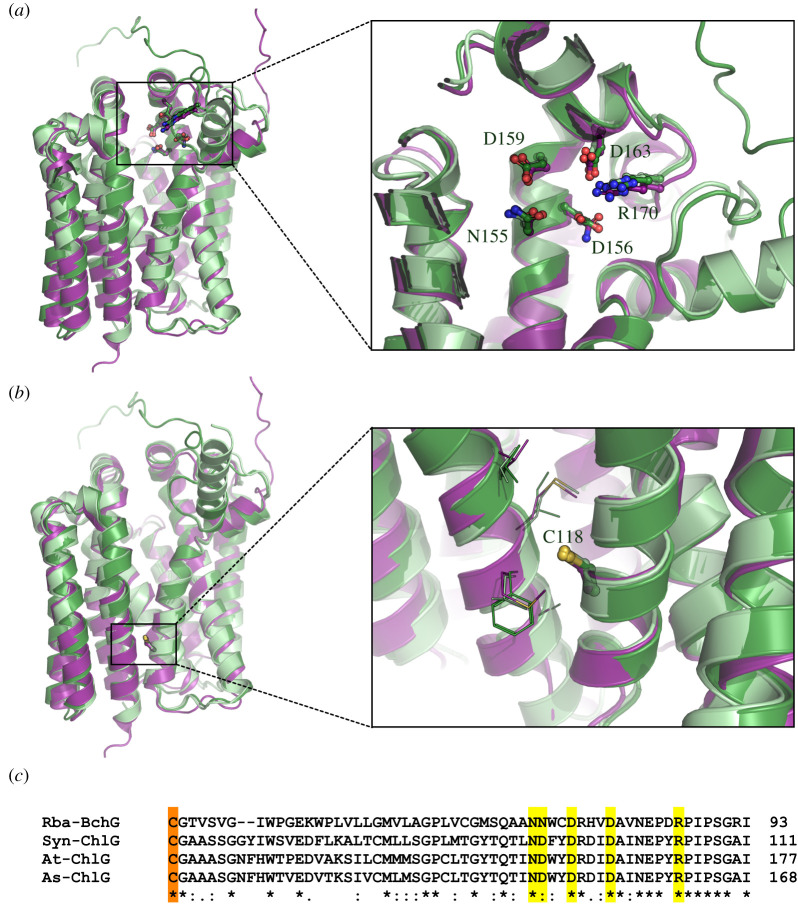
Highly conserved residues in (bacterio)chlorophyll synthases. Structural models of ChlG from *A. thaliana* (At-ChlG, dark green) and *Synechocystis* (Syn-ChlG, light green), and BchG from *Rba. sphaeroides* (Rba-BchG, purple), are displayed in the left-hand panels. Conserved residues of interest are rendered in ball and stick format. Oxygen, nitrogen and sulfur atoms are coloured red, blue and yellow, respectively. (*a*) An expanded image of the acidic NDXXDRXXDXXXXXXR motif shows close spatial residue alignment for each structure. (*b*) An expanded image of the C118 position. Nearby residues that may participate in hydrophobic packing but have not been identified as critical for protein function are shown in wire format. Residue numbering corresponds to the primary sequence of *A. thaliana* ChlG. (*c*) Partial sequence alignment of Rba-BchG, Syn-ChlG, At-ChlG and the *A. sativa* ChlG (As-ChlG); residues from the NDXXDRXXDXXXXXXR motif (depicted in panel (*a*)) are highlighted in yellow and the conserved cysteine (depicted in panel (*b*)) in orange.

The acidic NDXXDRXXDXXXXXXR motif predicted to coordinate the tetrapyrrole substrate is located with near-identical geometry in the cleft of each protein; the only notable difference is the presence of Asn in the *Rba. sphaeroides* structure (position 72) rather than the equivalent Asp residues in the Chlide *a*-binding enzymes (figure 6*a*). The highly conserved Cys118 in *A. thaliana* aligns closely to the corresponding Cys residues in the *Rba. sphaeroides* and *Synechocystis* models (figure 6*b*). This residue resides outside the putative active site at the interface between the two diverging helices that form the cleft opening. The models do not provide any indication of disulfide bond formation; instead, this residue appears to pack among other hydrophobic residues (V375, I378, F379 and A382 in *A. thaliana*) and its essential role presumably derives from hydrophobic packing and van der Waals interactions.

## 10. The role of chlorophyll synthase in coordination of chlorophyll biosynthesis, thylakoid biogenesis and photosystem repair in oxygenic phototrophs

Due to its position at the end of the Chl biosynthesis pathway, ChlG is thought to be important for co-regulation of Chl production and pigment delivery to Chl-binding proteins [20,148]. As such, a mismatch in the rate of Chl biosynthesis and the synthesis of Chl-binding proteins could result in an excess of free Chl to the detriment of the cell [149]. *De novo* production of Chl by ChlG is essential for the stable accumulation of Chl-binding apoproteins such as P700, CP47, CP43 and D2 [53,54,150–158], without which photomorphogenesis is delayed [40]. Although the accumulation of Chl and Chl-binding proteins are positively correlated [153,159–162], it is likely that the binding of Chl to these apoproteins is required for their stability and that the onset of Chl biosynthesis does not promote translation of Chl-binding photosystem subunits [54,154,155,163–165]. Furthermore, the binding of Chl to apoproteins appears to specifically require that Chl originates from *de novo* synthesis by ChlG; addition of Chlide and PPP to a ChlG-containing etioplast fraction resulted in accumulation of P700, CP47, CP43 and D2, whereas direct addition of Chl did not ([51]; L. Eichacker 2022, personal communication). This suggests ChlG participates in an as-yet uncharacterized mechanism in which newly synthesized Chl is delivered to nascent Chl-binding polypeptides co-translationally. A pause in translation by the ribosome may allow time for Chl binding [20,166–168], although this pause was still observed in Chl-free plants, so is not induced by Chl itself [165,167]. Nevertheless, the co-purification of *Synechocystis* ChlG with the membrane protein insertase YidC suggests interaction between the two proteins [20]. YidC is known to aid protein folding [169] and participate in the correct integration of photosystem polypeptides into thylakoid membranes [170–176], which could indicate direct Chl handover from ChlG to assembling photosystem apoproteins [20], although this has yet to be conclusively demonstrated. Other factors, such as the numerous auxiliary proteins that aid photosystem assembly, may also be involved in Chl handover (reviewed in [141]).

ChlG may also have an indirect role in the regulation of earlier enzymes in the Chl biosynthesis pathway and Chl-binding apoproteins by exerting control over Chl accumulation. In rice, mutation of *chlG* resulted in accumulation of Chl biosynthesis intermediates and the suppression of nuclear-encoded PSII genes, while plastid-encoded genes were unaffected, suggesting feedback regulation of nuclear gene expression by Chl [40]. Lack of Chl also resulted in reduced expression of the gene HEMA1, involved in producing glutamyl tRNA from which tetrapyrrole compounds, including Chl and heme, are synthesized [40]. This may be to prevent accumulation of Chl/heme precursors that can generate damaging reactive oxygen species upon exposure to light. Similarly, a heat-sensitive *chlG* mutant of *A. thaliana* that accumulates Chlide at increased temperature also had reduced levels of the Lhcb1 antenna protein [177].

The level of ChlG is also directly involved in feedback-control of Chl biosynthesis. Shalygo *et al*. [178] showed that *chlG* overexpression and knockdown lines of tobacco (*Nicotiana tabacum*) had increased and decreased transcript levels of magnesium chelatase, respectively. Magnesium chelatase catalyses the first dedicated and rate-limiting step in Chl biosynthesis and is therefore a valid target for control of the rate of Chl production. Aminolevulinic acid (ALA) synthesis was also decreased but Chlide accumulation was not observed, suggesting a mechanism for the prevention of Chl precursor build-up, which would have detrimental consequences for the cell. Conversely, overproduction of ChlG increased the metabolic flux towards Chl biosynthesis by increasing synthesis of ALA, enhancing magnesium chelatase activity and the accumulation of light-harvesting complexes.

In addition to a role in thylakoid biogenesis, ChlG is also postulated to function in the recycling of Chl molecules released from photodamaged protein complexes. Photodamage, in particular to PSII [179], necessitates a constant repair cycle where complexes are disassembled and damaged subunits are replaced by newly synthesized ones (reviewed in [156,157,180–184]). During PSII repair, Chl is released from the damaged subunits and recycled, firstly by removal of the tail, and then reintegration of the resulting Chlide and phytol (converted to PPP) back into the *de novo* Chl biosynthesis pathway [177,185,186]. A *chlG* mutant that is destabilized at temperatures ≥40°C accumulated Chlide in the dark, which must originate from de-esterification of Chl released from damaged photosystems rather than from *de novo* biosynthesis, which requires the light-dependent reaction of POR. This suggests that the pathway of Chl recycling uses ChlG for the re-esterification of Chlide and funnelling of the pigment back to the photosystem repair machinery for reintegration into PSII [177].

## 11. Chlorophyll synthase forms a complex with high-light inducible proteins in cyanobacteria

In cyanobacteria, the Chl-binding proteins of PSI and PSII are synthesized on TM-bound ribosomes and co-translationally bound to Chl prior to assembly into functioning photosystems [187]. Evidence to support the hypothesis that ChlG is involved in Chl delivery to nascent photosystem polypeptides emerged from co-immunoprecipitation assays using tagged *Synechocystis* ChlG. Recovery of a pigmented protein complex resulted in identification of high-light inducible proteins D and C (HliD/HliC), the PSII assembly factor Ycf39 and the membrane insertase YidC, along with Chl *a* and carotenoids [20,47]. ChlG binds tightly to HliD in a ChlG-HliD ‘core’ complex, with Ycf39 and YidC more-loosely associated and subsequently found to be relatively minor components of the complex [20,45]. There is also evidence that BchG interacts with YidC, YajC (an inner membrane protein that associates with the Sec machinery) and LhaA (the assembly factor for the light-harvesting 1 complex) in *Rba. sphaeroides* [188].

YidC is presumed to mediate ChlG integration into the thylakoid membrane and possibly facilitate co-translational insertion of Chl into photosystem apoproteins during thylakoid biogenesis [20], as discussed above. The peripheral membrane protein Ycf39 is a member of a family of short-chain alcohol dehydrogenases but has not been shown to have any catalytic function [20,189] and its role within the ChlG complex remains unclear. Ycf39 dissociates from the ChlG complex under high-light stress and can form a separate complex with HliD/HliC, which are involved in photoprotection of early PSII assembly intermediates and Chl recycling [45,189]. These different protein assemblies may represent dynamic sub-populations of the larger ChlG-HliD-HliC-Ycf39 complex that serve distinct functions in response to cellular stress.

HliD and HliC are two of the four HLIPs produced in *Synechocystis* [190] and are single-helix integral membrane proteins thought to be the ancestors of plant LHC proteins [191–194]. HLIPs possess a highly conserved Chl *a* binding domain and significantly increase the half-life of Chl in *Synechocystis*. They are also hypothesized to scavenge the Chl molecules released during the repair of damaged PSII [195–197]. Although not essential for cell survival [198], HLIPs function to protect the photosystems from photooxidative damage induced by excess light [190,191,197,199–201].

HliC and HliD associate into their respective homodimeric complexes *in vivo*; the former binds four Chl and two *β*-carotene pigments [202], whereas the latter binds six Chls and two *β*-carotenes [47,198]. The binding of HliD/HliC to ChlG accounts for the presence of *β*-carotene and the majority of the Chl within this complex [47]. HliD binds these pigments in a configuration that enables quenching of harmful Chl triplet excited states by *β*-carotene [203,204]. This quenching behaviour has been demonstrated within the ChlG-HliD complex, and so HliD is hypothesized to provide photoprotection to ChlG [47]. Incorporation of HliD into the ChlG complex is dependent upon zeaxanthin [46], which probably binds at the transmembrane interface between the two integral membrane proteins and acts as a ‘molecular glue’, increasing the strength of their interaction. Preventing the formation of the ChlG-HliD complex, either by removal of xanthophylls or deletion of *hliD* [20,46], resulted in accumulation of Chlide, indicating perturbed ChlG function. Deletion of *hliD* also reduces the quantities of ChlG that can be purified by FLAG immunoprecipitation, suggesting that HliD binding is required to stabilize the enzyme [20,46]. Despite this, the exact role of HliD within the ChlG complex has yet to be elucidated, and the role of HliC also requires further study.

Heterologous production of plant (*A. thaliana*) and algal (*Chlamydomonas reinhardtii*) ChlG homologues in *Synechocystis* allowed deletion of the otherwise essential native *chlG*, demonstrating that the eukaryotic enzymes are functional in the cyanobacterial host. The plant-enzyme producing strains did not display an obvious phenotype; however, they did not co-purify with HliD, Ycf39 or carotenoids [45], which suggests that they do not form such a complex *in vivo* or that the complex is less stable than the one formed by the cyanobacterial enzyme. Despite this, formation of equivalent ChlG complexes in plants and algae cannot be ruled out. Homologues of HLIPs are present in plants; in *A. thaliana*, one-helix protein 2 (OHP2) is the closest homologue to HliD [205]. Although a specific interaction of OHP2 and ChlG has not been reported, this protein has been shown to form a complex with OHP1 and the plant homologue of Ycf39, HCF244. This complex was implicated in Chl delivery to PSII apoproteins, resembling the Ycf39-HliD/HliC complex in *Synechocystis* [206–209]. Plants also possess light-harvesting-like (LIL) proteins, of which LIL3 is involved in the latter stages of Chl biosynthesis, interacting with both ChlP and POR [210–212]. Mork-Jansson *et al*. [211] demonstrated an interaction between LIL3 and ChlG in barley via a split-ubiquitin assay; however, Hey *et al*. [210] did not find such an interaction in *A. thaliana*.

## 12. Concluding remarks

Since the discovery of chlorophyll synthase activity over 100 years ago, a huge amount of progress has been made in characterizing this enzyme and its essential role in Chl biosynthesis, one of the most productive biological pathways on Earth. Similarly, our understanding of the BchG and BchK homologues required for BChl biosynthesis, and the tail-reducing ChlP/BchP enzymes, has also advanced. However, several important questions remain unanswered. While advances in protein structure prediction have improved our ability to model these enzyme structures, a key goal is the acquisition of *bona fide* high-resolution structures of the enzymes in order to help to elucidate their mechanisms of action and differing substrate specificities. Further characterization of the *in vivo* interactions between the synthases and ChlP/BchP and/or other enzymes in the (B)Chl biosynthesis pathways is also needed to determine if (B)Chl-biosynthesis complexes are formed to allow efficient substrate channelling from the soluble enzymes earlier in the pathway to the membrane-integral synthase. The role of the ChlG-HLIP complex identified in cyanobacteria, and whether analogous complexes exist in higher oxygenic phototrophs, are other avenues for future study. Furthermore, the exact role of ChlG in the handover of *de novo* synthesized Chl pigments to the photosystem assembly apparatus, and in the recycling of Chl released from damaged photosystems, will further our understanding of the interface between pigment biosynthesis and the assembly and repair processes. Answering these questions will help further our understanding of Chl metabolism in oxygenic phototrophs, a process essential for sustaining the majority of Earth's food chains, and aid our manipulation of photosynthesis, an endeavour that holds promise for the production of high-value biomolecules and increasing the yields of crops to feed the growing population.

## Data Availability

The model structures of At-ChlG, Syn-ChlG and Rba-BchG presented in this manuscript were generated from UniProt entries Q38833, Q55145, Q9Z5D6, respectively, and are available to download in the electronic supplementary material [213]. Since submission of this review, AlphaFold coordinate files for At-ChlG (identifier: AF-Q38833-F1) and Rba-BchG (identifier: AF-Q9Z5D6-F1) have been linked to the UniProt entries; these models align very closely (RMSD < 0.15) with the structures presented here.
